# Rare genetic variants impact muscle strength

**DOI:** 10.1038/s41467-023-39247-1

**Published:** 2023-06-10

**Authors:** Yunfeng Huang, Dora Bodnar, Chia-Yen Chen, Gabriela Sanchez-Andrade, Mark Sanderson, Biogen Biobank Team, Christopher D. Whelan, Paola Bronson, David Sexton, Sally John, Eric Marshall, Mehool Patel, Saranya Duraisamy, Timothy Swan, Denis Baird, Susan Eaton, Jake Gagnon, Feng Gao, Cynthia Gubbels, Varant Kupelian, Kejie Li, Dawei Liu, Stephanie Loomis, Helen McLaughlin, Adele Mitchell, Benjamin Sun, Jun Shi, Katherine G. Meilleur, Matthew E. Hurles, Sebastian S. Gerety, Ellen A. Tsai, Heiko Runz

**Affiliations:** 1 Research and Development, Biogen Inc., Cambridge, MA USA; 2 Wellcome Sanger Institute, Hinxton, Cambridge, United Kingdom

**Keywords:** Genetic association study, DNA sequencing, Skeletal muscle

## Abstract

Muscle strength is highly heritable and predictive for multiple adverse health outcomes including mortality. Here, we present a rare protein-coding variant association study in 340,319 individuals for hand grip strength, a proxy measure of muscle strength. We show that the exome-wide burden of rare protein-truncating and damaging missense variants is associated with a reduction in hand grip strength. We identify six significant hand grip strength genes, *KDM5B*, *OBSCN*, *GIGYF1*, *TTN*, *RB1CC1*, and *EIF3J*. In the example of the titin (*TTN)* locus we demonstrate a convergence of rare with common variant association signals and uncover genetic relationships between reduced hand grip strength and disease. Finally, we identify shared mechanisms between brain and muscle function and uncover additive effects between rare and common genetic variation on muscle strength.

## Abstract (web summary)

Here, the authors provide an exome study of hand grip strength, a proxy of generalized muscle strength. They identify six exome-wide significant genes, with links to disease, and additivity of rare and common genetic variant effects on muscle strength.

## Introduction

Muscle strength is a key measure of physical ability and overall health and when reduced is associated with adverse health outcomes including disability and mortality^1^. Muscle strength is highly heritable (*h*^2^ ~ 0.4)^2^, but the underlying genetic architecture and mechanisms remain unclear. Hand grip strength is a reliable proxy measure of general muscle strength with genome-wide associations studies (GWAS) having identified >180 common-variant-based loci^3^. No study has yet systematically analyzed the contribution of rare protein-coding variation to hand grip strength. Here, we leverage whole exome sequencing in 340,319 UK Biobank (UKB)^4^ participants to uncover genes in which rare coding variants are associated with hand grip strength.

## Results

### Global rare coding variant burden impacts hand grip strength

**Fig. 1:**
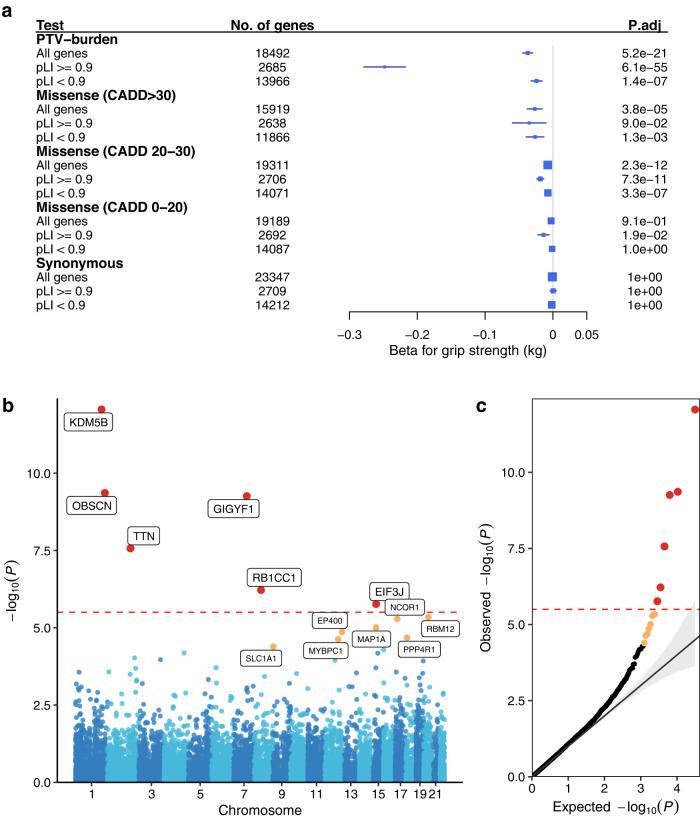
Genome-wide rare variant burden associations with hand grip strength. **a** Rare variant burden associations with hand grip strength stratified on PTV, very damaging missense (CADD > 30), damaging missense (CADD 20–30), other missense (CADD 0–20), and synonymous variants. Associations were tested for all, LoF intolerant (pLI > 0.9) and LoF tolerant (pLI < 0.9) genes, respectively. *P*-values were derived from two-sided *t*-tests of linear regression coefficients. A Bonferroni correction was used to adjust for multiple testing. No. of genes: number of genes included in each rare variant burden association test; P.adj: Bonferroni-adjusted *P*-values. **b**, **c** Manhattan and QQ plot of gene-based PTV-burden associations with hand grip strength. *P*-values were derived from two-sided *t*-tests of linear regression coefficients. Bonferroni and false discovery rate (FDR) correction were used to adjust for multiple testing. The Red dashed line indicates the Bonferroni significance threshold, the red circle indicates significant genes that passed the Bonferroni threshold, and orange circles indicate genes with FDR < 0.05. Source data are provided in Supplementary Data 1 and 2.

We first assessed globally the impact of rare coding variant burden on hand grip strength. Fifteen burden tests were conducted based on predicted effects of rare coding variants (protein-truncating (PTV), missense, or synonymous), and further on deleteriousness score for missense variants (Fig. 1a, Supplementary Data 1). When stratified by pLI (probability of loss-of-function (LoF) intolerance)^5^, the strongest effect on hand grip strength was observed for PTV-burden from LoF-intolerant genes (pLI ≥ 0.9, beta = −0.25 kg, *p* = 4.05 × 10^−56^). Significant associations (*p* < 0.05/15 = 0.0033) with reduced hand grip strength were further observed for PTV-burden in LoF-tolerant genes, very damaging and damaging missense variant burden.

### Rare variant tests identify six hand grip strength genes

Next, we conducted gene-level association analyses to identify individual genes in which a burden of PTV or missense variants affect hand grip strength. For six autosomal genes (*KDM5B*, *OBSCN*, *GIGYF1*, *TTN*, *RB1CC1*, *EIF3J*), PTV-burden showed significant association with hand grip strength after Bonferroni correction (*p* < 3.2 × 10^−6^) (Fig. 1b, Supplementary Data 2–4). A sex-stratified analysis showed consistent associations between males and females (Supplementary Data 2). An additional X-chromosome analysis identified no significant PTV or missense burden associations with hand grip strength after multiple testing corrections (Supplementary Data 5–7).

**Fig. 2:**
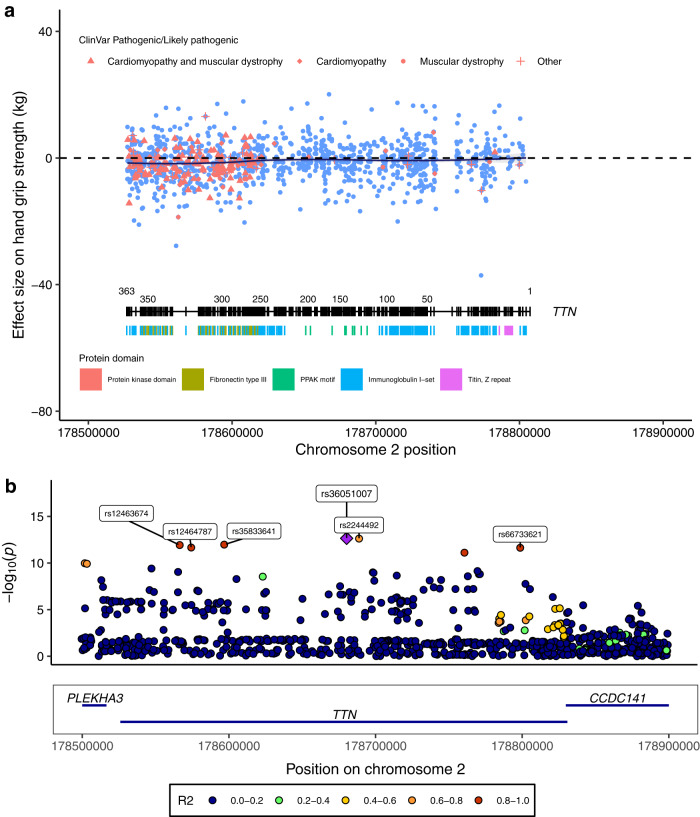
Converging rare and common variant associations of *TTN* with hand grip strength. **a** Single PTV association of *TTN* with hand grip strength. The average effect sizes of each PTV in *TTN* were plotted against their genomic positions, with exon numbers and protein domains visualized at the bottom. ClinVar pathogenic/likely pathogenic PTVs were colored in red with different shapes indicating their respective associated disease phenotypes. **b** hand grip strength GWAS locus plot of *TTN*. *P*-values were derived from two-sided *t*-tests implemented in regenie. The purple diamond indicates the sentinel variant at the *TTN* GWAS locus. SNP IDs label the top 95% credible set identified by SuSiE. Colors indicate different levels of LD with the sentinel variant. Source data are provided in Supplementary Data 10 and the Source Data file.

Notably, two genes identified were giant sarcomeric proteins titin (*TTN*) and obscurin (*OBSCN*), which are essential in maintaining the structure and function of striated muscle, with mutations in *TTN* causing Mendelian diseases affecting skeletal and cardiac muscle (OMIM #188840). Totally, 3925 *TTN* PTV carriers in UKB showed an average of −0.61 kg reduction of hand grip strength relative to non-carriers (*p* = 2.7 × 10^−8^, Fig. 2a). *TTN* PTV burden was also associated with the risk of cardiomyopathy, atrial fibrillation, and reduced muscle mass (Supplementary Fig. 1, Supplementary Data 8; for findings on other genes, see Supplementary Discussion). For *TTN*, *OBSCN*, and *RB1CC1*, we identified non-canonical transcripts that are primarily expressed in the skeletal muscle tissue based on GTEx data^6^. PTV-burden testing on these skeletal muscle transcripts showed largely consistent associations with tests based on canonical isoforms (see Supplementary Note, Supplementary Data 9).

We next assessed whether also common variants (minor allele frequency > 0.01) in *TTN* were associated with hand grip strength by conducting a GWAS in UKB followed by a gene-based analysis using MAGMA (v1.6)^7^. Indeed, *TTN* showed a significant gene-based association (*p* = 5.5 × 10^−8^) revealing that both rare and common genetic variants in *TTN* impact muscle strength. We utilized the rare PTV association results of *TTN* to finemap the *TTN* locus. Statistical finemapping identified a top 95% credible set of six variants including the damaging missense variant rs12463674 (CADD = 22.2, NP_001254479.2:p.Ile26225Thr) in high linkage disequilibrium (LD) with the sentinel SNP rs36051007 (r^2^ = 0.97, Fig. 2b) proposing rs12463674 as the causal variant for hand grip strength at this locus. Notably, we observed prominent clustering and larger hand grip strength effect sizes of ClinVar annotated pathogenic/likely pathogenic PTVs at exons 260–360 encoding for titin’s A-band fibronectin type III-immunoglobulin domains (Fig. 2a, Supplementary Data 10). This is consistent with this region being evolutionally highly conserved and of reported relevance to disease^8^. It further proposes that considering rare and common variant associations jointly may assist clinical variant interpretation. To expand beyond *TTN* and systematically investigate for potential overlap between our rare PTV-burden association results and previous GWAS findings, we extracted 236 common lead variants reported in the GWAS Catalog for “grip strength measurement” (EFO_0006941) (Supplementary Data 11) and mapped all protein-coding genes within 500 kb of these variants. However, none of the 1366 genes meeting these criteria showed a significant rare PTV-burden association with hand grip strength after Bonferroni correction (Supplementary Data 12). This indicates that at current sample sizes, common and rare genetic architecture for hand grip strength remains largely distinct.

### PTV-burden tests yield a genetic link between muscle and brain

**Fig. 3:**
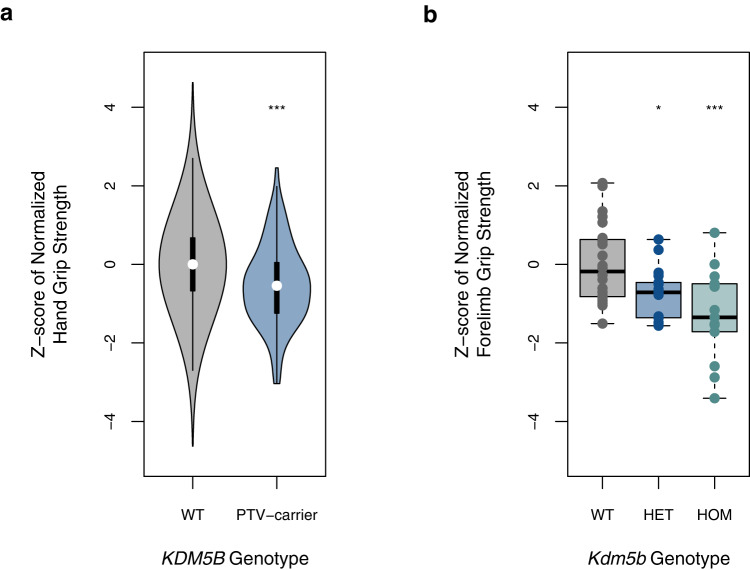
*KDM5B* loss-of-function causes a dose-dependent reduction in hand grip strength in humans and mice. **a** Normalized hand grip strength stratified for *KDM5B* PTV carrier status in *N* = 340,319 independent UK Biobank participants. Hand grip strength was normalized against standing height, then residualized on age, sex, and PCs. *Z*-scores were generated through inverse-rank normalization, then compared between *KDM5B* PTV carriers vs. non-carriers (WT). *P*-values were derived using two-sided *t*-tests. ***P = 2.86 × 10^−12^. **b** Forelimb grip strength of mice stratified on *Kdm5b* genotype in *N* = 55 biologically independent animals. FGS was normalized against femur length, then corrected for the cohort effect. *Z*-scores calculated from residuals were then compared across heterozygous (HET) and homozygous (HOM) *Kdm5b* mutant and wild-type (WT) animals. *P*-values were derived using two-sided *t*-tests. *P = 0.016; ***P = 0.0002; center line: median; box limits: upper and lower quartiles; whiskers: minimum or lower quartile minus 1.5 times IQR and maximum or upper quartile plus 1.5 times IQR; Source data are provided in the Source Data file.

Hand grip strength GWAS loci are enriched for skeletal muscle and central nervous system genes^9^. Consistently, hand grip strength PTV associations were enriched for the brain- and muscle-expressed genes, as well as neurodevelopmental and muscle-related pathways (Supplementary Fig. 5, Supplementary Data 13). A tight link between brain and muscle function is further supported by our recent finding that PTV-burden in two of the six novel hand grip strength genes, *KDM5B* and *GIGYF1*, is also associated with measures of cognitive function, with *KDM5B* PTVs showing dose-dependent effects in humans^10^ and mice^11^. As for cognitive function parameters, *Kdm5b* mutant mice^12^ are characterized by reduced forelimb strength, with heterozygote mice showing intermediate effect sizes to homozygote and wildtype mice, respectively (Fig. 3, Supplementary Figs. 3 and 4). We conducted sensitivity analyses correcting hand grip strength PTV-burden associations for educational attainment and reaction time as covariates. Such adjustment had only a minimal impact on the top findings for hand grip strength (Supplementary Data 14), suggesting that the respective impact of rare variants on cognitive and muscle function is likely related, yet largely independent. Beyond putative direct roles in the central nervous system, exome studies have linked *KDM5B* and *GIGYF1* variants to blood levels of insulin-like growth factor 1 (IGF-1)^13^, suggesting that loss of function of these genes might impair skeletal muscle function through perturbing IGF-1 signaling (Supplementary Discussion).

### Additivity of rare and common variants on hand grip strength

Finally, we assessed the interplay between common and rare disease-relevant genetic variation on hand grip strength. A polygenic risk score for hand grip strength (PRS_HGS_) was constructed for 38,118 carriers of PTVs in 199 Mendelian neuromuscular disease genes as well as 38,118 randomly selected non-carriers in UKB (Supplementary Data 15). We observed a significant reduction in hand grip strength for carriers of PTVs in autosomal-dominant neuromuscular disease genes compared to non-carriers (beta = −0.196 kg, *p* = 0.0097), while no reduction was observed for carriers of PTVs in autosomal-recessive neuromuscular disease genes. Likewise, an increase in hand grip strength was significantly associated with per-SD higher PRS_HGS_ (beta = 0.954 kg, *p* = 1.5 × 10^−294^). Notably, high PRS_HGS_ mitigated the reduction in hand grip strength for PTV carriers of autosomal-dominant neuromuscular disease genes, with the effect between PRS_HGS_ and rare PTV-burden in Mendelian neuromuscular disease genes being additive (Supplementary Fig. 6, Supplementary Data 16).

## Discussion

In summary, we conducted the largest rare-variant association study for muscle strength to date. We identified six genes associated with hand grip strength and support a role for CNS and muscle-related genes and pathways in muscle strength. For *TTN*, we highlight how rare PTVs can assist in the finemapping of a GWAS locus and how rare variants ascertained from a biobank population expand the phenotypic spectrum of a medically actionable gene^8^. While our exome study is not yet powered to unravel individual hand grip strength loci beyond *TTN* where common and rare variant signals converge, we demonstrate additive effects between rare coding variation in Mendelian neuromuscular disease genes and PRS_HGS_. Our results further indicate that at least some of the associations identified might be influenced by the effects of muscle-specific rather than canonical transcripts of the respective genes. Future studies will need to assess putative tissue-specific effects more systematically and further refine our understanding of the genetic basis of human disease resulting from that.

## Methods

### The UKB and whole-exome sequencing

The UKB is a large prospective population-based study with over half a million participants recruited across the UK^4^. Phenotypic data collected from each participant includes survey measures, electronic health records, self-reported health information, and other biological measurements. The participants have diverse genetic ancestries and overrepresented familial relatedness. Whole-exome sequencing (WES) data from UKB participants was generated by the Regeneron Genetics Center as part of a collaboration between AbbVie, Alnylam Pharmaceuticals, AstraZeneca, Biogen, Bristol-Myers Squibb, Pfizer, Regeneron, and Takeda. The WES production and quality control (QC) is described in detail in Van Hout et al.^14^. As of November 2020, we obtained QC-passed WES data (“Goldilocks” set) from 454,787 samples in the UKB. Analyses in this study were conducted under UKB Approved Project number 26041. Ethical protocols are provided by the UKB Ethics Advisory Committee (https://www.ukbiobank.ac.uk/learn-more-about-uk-biobank/about-us/ethics).

### Variant annotation

We annotated variants identified through WES by Variant Effect Predictor (VEP) v96 with genome build GRCh38^4^. Variants annotated as stop-gained, splice site disruptive, and frameshift variants were further assessed Loss-Of-Function Transcript Effect Estimator (LOFTEE), a VEP plugin. LOFTEE implements a set of filters to remove variants that are unlikely to be disruptive. Those variants labeled as “low-confidence” were filtered out, and we kept variants labeled as “high-confidence”. Variants annotated as missense variants were then annotated by CADD score^15^, which prioritized damaging missense variants. All predicted variants were mapped to GENCODE canonical transcripts^4^. In total, we identified 714,260 predicted rare PTVs, 6,675,884 missense variants, and 3,884,581 synonymous variants with minor allele frequency <0.1%. As a sensitivity analysis for the significant gene-level findings, we also examined transcripts that are primarily expressed in the skeletal muscle, to explore potential isoform-specific signals (Supplementary Note).

### Phenotyping of hand grip strength

Hand grip strength was measured for both left and right hands using a hydraulic hand dynamometer while seated. A detailed protocol can be found at the UKB data showcase site. Out of 409,559 UKB participants of European ancestry (Data-Field 22006), we removed individuals with disease diagnoses that can potentially confound hand grip strength measurements including COPD (*N* = 2616), brachial plexus disorders (*n* = 50), or history of injuries in elbow, forearm, wrist and hand (*n* = 7608). We also conducted sensitivity analyses to assess the impact of several other diseases including osteoarthritis, rheumatoid arthritis, rhizarthrosis, osteoporosis, Dupuytren’s contracture, and cancer. (Supplementary Note and Supplementary Data 17 and 18). Due to the large impact of body size on hand grip strength, we restricted our analysis to samples with non-missing and normal body weight (30–200 kg).

We also conducted a thorough sensitivity analysis to evaluate which anthropometric covariates to include in order to best capture potential genetic signals, not driven-by body size or obesity-related traits. In such analysis, we tested standing height, BMI, as well as whole body fat mass as candidate covariates for hand grip strength GWAS. Adjustment of standing height had a large impact on the number of significant GWAS findings but further adjustment of BMI or whole-body fat mass gave almost identical results. Therefore, we decided to include standing height as the only anthropometric covariate in our final genetic analysis to avoid unnecessary correction. We also conducted a sensitivity analysis to assess the impact of skeletal muscle mass adjustment on the PTV-burden associations we identified for hand grip strength, the significant findings for hand grip strength were consistent after further adjustment of whole-body muscle mass. (Supplementary Note and Supplementary Data 19) Samples with grip strength measured for at least one hand were kept in the analysis. The maximum grip strength between two hands was taken as the final outcome for genetic analysis. In total, 340319 samples past QC and filtering were included in the exome analysis.

### Whole-exome and gene-level burden test

We grouped protein-coding genes by pLI (v2.1.1)^5^, into LoF intolerant (pLI ≥ 0.9) set and LoF tolerant (pLI < 0.9) set. We annotated rare variants by functional consequences into three types, protein-truncating, missense, and synonymous. Missense variants were further annotated by CADD score^15^ and stratified into 3 groups by predicted deleteriousness, CADD > 30, 30 ≥ CADD > 20, 20 ≥ CADD > 0. In total, we tested hand grip strength association with 15 sets of variants: PTV, CADD > 30, 30 ≥ CADD > 20, 20 ≥ CADD > 0 and synonymous variants in pLI ≥ 0.9 genes; PTV, CADD > 30, 30 ≥ CADD > 20, 20 ≥ CADD > 0 and synonymous variants in pLI < 0.9 genes; and PTV, CADD > 30, 30 ≥ CADD > 20, 20 ≥ CADD > 0 and synonymous variants in all genes. Rare alleles of the same variant category on each gene were aggregated into a gene-level burden. The summation of the burden on genes in each gene set was the whole-exome burden.

For the whole-exome burden test, we applied linear regression (“lm” function in R v3.6.1) by fitting whole-exome burden to maximum hand grip strength as the continuous outcome. In the model, we controlled for population structure with top 20 PCs, age, sex, age-squared, age × sex, age-squared × sex, standing height, standing height-squared, standing height × sex, standing height-squared × sex. We also applied the “regenie”^16^ methods to construct genome-wide predictors of hand grip strength using genotype array data and included them as model offset to account for additional genetic confounding. We defined a significant threshold at *P* < 0.0033 (0.05/15) for the whole-exome burden tests.

For the gene-level burden test, we fitted linear regression models by regressing hand grip strength on the rare variant burden of each gene. We restricted to PTV and damaging missense (CADD > 20) burden for gene-level burden test, based on significant associations in the whole-exome burden tests. We included the same covariates as described above and the corresponding leave-one-chromosome-out (LOCO) predictors constructed by “regenie”^16^ (v2.0.1) as the model offset. We excluded genes with less than 10 carriers for PTV or damaging missense burden. In total, we tested 15,786 autosomal genes for PTV, 17,557 autosomal genes for very damaging missense variants (CADD > 30) and 11,370 autosomal genes for damaging missense variants (CADD 20–30). Bonferroni correction was applied to declare statistical significance for each category, i.e., *P* < 3.2 × 10^−6^ for PTV, *P* < 2.8 × 10^−6^ for very damaging missense variants, and *P* < 4.4 × 10^−6^ for damaging missense variants. After further exclusion of 417 participants with sex chromosome aneuploidy, a separate X-chromosome analysis was also conducted for PTV, very damaging missense variants and damaging missense variants of 541, 97, and 686 genes, respectively.

### GWAS of hand grip strength, MAGMA gene-based analysis, and fine mapping of TTN locus

To assess whether there are converging rare and common variant associations of *TTN* and *OBSCN* with hand grip strength, we also conducted a GWAS focusing on common variants with minor allele frequency >0.01 in the UKB. 366,307 UKB participants of European ancestry post-QC were included in the GWAS of hand grip strength, adjusted for age, sex, age-squared, age × sex, age-squared × sex, standing height, standing height-squared, standing height × sex, standing height-squared × sex, batch effects and top 20 PCs. “regenie” genome-wide predictors were included as model offset to account for additional genetic confounding. A MAGMA (v1.6) gene-based analysis was conducted using FUMA (v1.3.6, https://fuma.ctglab.nl/,) with the GWAS summary statistics to identify converging common variant associations for *TTN* and *OBSCN*. Statistical fine mapping of the *TTN* GWAS locus was conducted using SuSiE^17^ implemented in “susieR” package with default parameters.

### Systematic overlap analysis of previously reported hand grip strength-associated common variants and rare PTV-burden

We extracted a total of 269 associations reported for “grip strength measurement” in the GWAS Catalog (https://www.ebi.ac.uk/gwas/efotraits/EFO_0006941) and filtered to 236 associations with minor allele frequency >0.01. We then annotated all protein-coding genes within a ±500 kb window of these reported lead variants based on GENCODE annotations and identified 1787 protein-coding genes for these common variant loci (Supplementary Data 11). Totally, 1366 of these genes were included in our rare PTV-burden association analysis with hand grip strength. (Supplementary Data 12) We checked the significance of these associations.

### Gene-set PTV-burden analysis

We conducted a self-contained gene-set PTV-burden analysis to further capture the impact of rare PTV in particular gene sets on hand grip strength, including genes with elevated expression in different tissues as well as genes in certain ontology categories or biological pathways. Gene-set PTV-burden is constructed by summing the rare alleles of PTVs of all genes in each gene-set, association testing was carried out using linear regression (“lm” function in R) by fitting gene-set burden to maximum hand grip strength as the continuous outcome. In the model, we controlled for population structure with top 20 PCs, age, sex, age-squared, age × sex, age-squared × sex, standing height, standing height-squared, standing height × sex, standing height-squared × sex. We also applied the “regenie”^16^ methods to construct genome-wide predictors of hand grip strength using genotype array data and included them as model offset to account for additional genetic confounding.

### Genes with elevated expression in different tissues

We explored how rare PTV-burden of genes enriched in different tissues impact hand grip strength based on data from the Human Protein Atlas (HPA, http://www.proteinatlas.org). Based on data from the HPA, we extracted 10,992 genes that showed elevated expression in at least 1 of 36 different tissues including a range of 51 genes for smooth muscle to 2709 genes for the brain. We calculated the PTV burden for genes that showed elevated expression in each tissue. Rare alleles of the PTV on each gene were aggregated into a gene-level burden; the summation of the burden on genes was the gene-set burden. A false discovery rate <0.05 for 36 tissues was applied to declare significance for this analysis.

### Pathway-based gene-set PTV-burden

We also constructed pathway-based gene-set PTV-burden for 20,928 gene sets from MSigDB v7.2^18^ including 10,266 Gene Ontology (GO) terms (7569 biological processes, 1001 cellular components, 1696 molecular functions), 4493 Human Phenotype Ontology terms, 3354 curated chemical and genetic perturbation terms, and 2815 curated canonical pathways from BioCarta, KEGG, PID, Reactome, and WikiPathways^18^. Rare alleles of the PTV on each gene were aggregated into a gene-level burden; the summation of the burden on genes in each pathway-based gene set was the gene-set PTV burden. The pathway-based PTV-burden association with hand grip strength was assessed in a self-contained manner and a false discovery rate < 0.05 was used to declare statistical significance (Supplementary Data 13).

### Phenome-wide association study (PheWAS)

We performed a PTV-burden of *TTN* PheWAS across 3654 binary and 238 quantitative phenotypes. Each binary phenotype was either derived from an ICD10 diagnosis code in the UKB and mapped to a Phecode, or derived from self-reported illnesses, operation procedures, or medication use. We excluded phenotypes with less than 100 cases for binary phenotypes. We took a two-step approach to first test all gene-phenotype pairs by logistic regression and then performed Firth’s logistic regression for those gene-phenotype pairs that passed a significant threshold (*P* < 0.01). For quantitative phenotypes, we excluded phenotypes with fewer than 100 observations and phenotypes with less than 12 distinct values. For each phenotype, we removed individuals with phenotype values> 5 SDs from the sample mean. Burden testing was performed using linear regression on both the raw and inverse rank-based normal transformed quantitative phenotypes. We controlled for population structure with the top 20 PCs, age, sex, age-squared, age × sex, age-squared × sex in the PheWAS. We defined phenome-wide significant thresholds as *P* < 1.3 × 10^−5^ (0.05/3892).

### Polygenic risk score (PRS) analysis

To assess the interplay between common variants and rare PTVs for hereditary neuromuscular disease genes on hand grip strength, we first developed a PRS for hand grip strength in the UKB European samples excluding 38118 carriers of PTVs in 199 genes associated with Mendelian neuromuscular diseases as well as 38,118 randomly selected non-carriers. A GWAS of hand grip strength was then conducted using the rest of the UKB samples in the same manner as described above. A PRS for hand grip strength was then constructed based on the summary statistics using the PRS-CS method^19^ with default settings and no pre-specified phi value. LD reference panel was precomputed using 1000 Genomes Project phase 3 samples with European ancestry (available at https://github.com/getian107/PRScs). PRS of each chromosome for each individual in the validation set was computed by the “--score” function in PLINK 2.00 alpha, a linear combination of genotypes weighted by effect size estimates. The final PRS was then summed across chromosomes 1 to 22. We then tested the contribution of hand grip strength PRS and PTV-burden in Mendelian neuromuscular disease genes on hand grip strength in the UKB reserved samples (38,118 carriers of PTVs in 199 genes associated with Mendelian neuromuscular diseases as well as 38118 randomly selected non-carriers), as well as the interaction between PRS and PTV-burden. We binarized the PTV burden to carriers vs. non-carriers. To have a better understanding of the potentially different contributions of PTVs in Mendelian neuromuscular disease genes with dominant vs. recessive inheritance, we further categorized PTV carriers into carriers for autosomal dominant neuromuscular diseases vs. autosomal recessive neuromuscular diseases. (Supplementary Data 16) We then applied linear regression with hand grip strength as a continuous outcome:

1 $${{{{{\rm{HGS}}}}}} \sim 	{{{{{{\rm{PRS}}}}}}}_{{{{{{\rm{std}}}}}}}+{{{{{{\rm{PTV}}}}}}}_{{{{{{\rm{carrier}}}}}}-{{{{{\rm{dom}}}}}}}+{{{{{{\rm{PTV}}}}}}}_{{{{{{\rm{carrier}}}}}}-{{{{{\rm{rec}}}}}}}+{{{{{{\rm{PRS}}}}}}}_{{{{{{\rm{std}}}}}}}\times {{{{{{\rm{PTV}}}}}}}_{{{{{{\rm{carrier}}}}}}-{{{{{\rm{dom}}}}}}} \\ 	+ {{{{{{\rm{PRS}}}}}}}_{{{{{{\rm{std}}}}}}}\times {{{{{{\rm{PTV}}}}}}}_{{{{{{\rm{carrier}}}}}}-{{{{{\rm{rec}}}}}}}$$

HGS: hand grip strength (maximum); PRS_std_: standardized hand grip strength PRS; PTV_carrier-dom_: PTV carriers for genes of autosomal dominant Mendelian neuromuscular diseases; PTV_carrier-rec_: PTV carriers for genes of autosomal recessive Mendelian neuromuscular diseases;

In the model, we also controlled for population structure with top 20 PCs, age, sex, age-squared, age × sex, age-squared × sex, standing height, standing height-squared, standing height × sex, standing height-squared × sex.

### In vivo KDM5B experiment

#### Animals

The generation of a mouse Kdm5b loss of function allele (MGI:6153378), its breeding, and housing has been previously reported^10, 12^. Briefly, generation was through a CRISPR/CAS9 mediated deletion of coding exon 7 (ENSMUSE00001331577), leading to a premature translational termination due to a downstream frameshifted reading frame. Breeding of test cohorts was performed on a C57BL/6NJ background. Breeding, housing, and all experimental procedures with mice were approved by the Animal Welfare and Ethical Review Body of the Wellcome Sanger Institute and, conducted under the regulation of the UK Home Office license (P6320B89B), and in accordance with institutional guidelines. At 14–15 weeks of age, 24 wildtypes, 18 heterozygous, and 13 homozygous Kdm5b mutant male mice were tested, in two independent cohorts.

#### Grip strength measurement

A Grip Strength Meter (BIO-GT3 + MR; Bioseb, France) with a custom grid attachment (stainless steel, 9.8 × 11 cm, with 2 mm bars 0.85 cm apart, German Mouse Clinic, Neuherberg, Germany) was used to measure forelimb grip strength. Experimenters were blind to genotype. As a mouse grasped the grid with the forepaws, it was pulled off the grid, and the peak pull force in grams was recorded on a digital transducer. This was repeated three times for each mouse and the mean value was used. Grip strength normalization to body size was calculated as the mean grip strength divided by femur length.

#### X-rays

Half the mice were anesthetized with ketamine/xylazine (100 mg/10 mg per kg of body weight) and then placed in an MX-20 X-ray machine (Faxitron X-Ray LLC). Whole body x-radiographs were taken in a dorsoventral orientation. A second set of x-radiographs were generated from independent animals using post-mortem dissected legs. All images were analyzed and morphological abnormalities were assessed using Sante DICOM Viewer v7.2.1 (Santesoft LTD).

#### Statistical analysis (mouse data)

All statistical analyses of mouse data were performed using R. Data was first transformed to achieve normality, using Box–Cox transformation (MASS package, with lambda limit = [−2,2]). Testing for the genotype effect was performed using a double generalized linear model, dglm (dglm package). The cohort was used as a covariate for the Box–Cox transformation as well as the dglm test. For visualization purposes, residual values were calculated from the linear model, and *z*-scores relative to wild types were calculated.

### Reporting summary

Further information on research design is available in the Nature Portfolio Reporting Summary linked to this article.

## Supplementary information

Supplementary Information

Description of Additional Supplementary Files

Supplementary Data 1-19

Reporting Summary

## Data Availability

All phenotypic and genetic data for the UK Biobank are available to researchers under data access request from the UK Biobank. WES data from UK Biobank participants have been deposited with UK Biobank and are freely available to approved researchers via the UK Biobank Research Analysis Platform (https://www.ukbiobank.ac.uk/enable-your-research/research-analysis-platform). Data used for this study is under application 26041. Summary-level association results produced in the present study are contained in the Supplementary Data. Public datasets used in the present study include pLoF Metrics (https://storage.googleapis.com/gcp-public-data--gnomad/release/2.1.1/constraint/gnomad.v2.1.1.lof_metrics.by_gene.txt.bgz); Human Protein Atlas (https://www.proteinatlas.org/humanproteome/tissue/tissue+specific); CADD score (https://cadd.gs.washington.edu/download); MSigDB (https://www.gsea-msigdb.org/gsea/msigdb/). Source data are provided in this paper.

## Source data

Source Data

## Code availability

Computational codes used for analyses in the present study are available under the following link: https://github.com/yunfenghuang618/HGS_exome_paper.
