# Supplementary material for: Rare genetic variants impact muscle strength
Source: Nat Commun. 2023 Jun 10;14:3449. doi: 10.1038/s41467-023-39247-1 (PMC10257725; doi:10.1038/s41467-023-39247-1)
Supplement: Supplementary file 1 — Supplementary Information [file 41467_2023_39247_MOESM1_ESM.pdf]

Rare genetic variants impact muscle strength

Supplementary Discussion

We conducted gene-level PTV and missense-burden association analyses to identify individual genes in which a burden of rare coding variants affect hand grip strength. In total, 15,786 genes with at least 10 carriers of PTVs with MAF<0.001 were included in the gene-level burden analysis. We found 6 genes (*KDM5B*, *OBSCN*, *GIGYF1*, *TTN*, *RB1CC1* and *EIF3J*) for which PTV-burden showed significant association with hand grip strength after Bonferroni correction ($p < 3.2 \times 10^{-6}$). Seven additional genes showed a false discovery rate (FDR)<0.05 (Figure 2b, Supplementary Data 2). We did not identify missense-burden associations at the level of individual genes (Supplementary Data 3-4).

TTN was discussed in the main text.

OBSCN

Obscurin is another giant sarcomeric protein. It is the third member of the sarcomeric giant protein family that, along with titin and nebulin, represents the underlying backbone that guides the assembly of the sarcomere and maintains its stability over time in striated muscle. In adult striated muscles, obscurin shows a definitive localization pattern with a larger distribution at the M-bands, bridge titin and myomesin at the M-band.[1,2] Protein-truncating variants of *OBSCN* have been associated with hypertrophic cardiomyopathy[3], and more recently homozygous LoF variants in *OBSCN* were identified to cause severe recurrent rhabdomyolysis[4]. Intriguingly, we found a higher grip strength for 3,834 heterozygous *OBSCN* PTV-carriers compared to non-carriers of UKB (beta=0.69kg, $p=4.4 \times 10^{-10}$). No reduction in HGS was observed for 470 heterozygous carriers of the pathogenic LoF variants reported to cause severe recurrent rhabdomyolysis[4] (Supplementary Figure 2).

KDM5B

Lysine-specific demethylase 5B (KDM5B, also called JARID1B or PLU1) is a JmjC domain containing enzyme that removes methyl groups from tri- or di-methylated H3K4 (H3K4me3/2) to produce monomethylated H3K4 (H3K4me1).[5] KDM5B is overexpressed in breast-, prostate-, bladder-, lung- and melanoma cancer, and serves as a potential oncogene. KDM5B has been shown to interact with the HDAC complexes. It has been shown, that HDAC inhibition decreased the level of *KDM5B* expression.[6] Histone deacetylases (HDACs), which targets histone and non-histone proteins, is a major enzyme family that controls the biological process of histone deacetylation, hence HDACs are essential for gene expression, metabolic and physiological function of the skeletal muscle system. Recent studies have shed light on the role of HDACs in skeletal muscle metabolism, myogenesis, maintaining sarcomere homeostasis, mitochondria remodeling, etc.[7] A previous report also demonstrated a potential regulatory role in glucose homeostasis and metabolism for KDM5B,[8] with KDM5B*Kdm5b* KO mice showing altered body composition and reduced IGF-1 levels, consistent with previously reported rare variant burden associations of *KDM5B* with blood IGF-1 levels in the UK Biobank.[9] *KDM5B* also plays an important role in early embryonic development and mutations in *KDM5B* lead to development delay and intellectual disability.[10] Given the correlation observed between hand grip strength and cognitive function[11] it is likely that part of the association between *KDM5B* PTV-burden and hand grip strength can be explained by cognitive deficits. However, our sensitivity analysis controlling for baseline education level or reaction time had minimal impact

on the association signal suggesting other uncaptured developmental mechanisms through which the brain-related genes might impact muscle strength.

GIGYF1

GIGYF1 encoding for GRB10-interacting GYF protein 1 can modulate insulin-like growth factor 1 receptor (*IGF-1R*) signaling pathway.[12] Upon the binding of IGF-1 to the IGF-1R, several important signaling pathways get activated, such as the PI3K/Akt-, the Akt/mTOR- and the GSK3β pathways. There is also a crosstalk between IGF-1 and myostatin signaling pathways. The IGF-1 and its receptor are key regulators of both anabolic and catabolic pathways.[13] It has been recently reported that loss-of-function variants in *GIGYF1* associated with risk of type II diabetes and increased glucose levels.[14]

RB1CC1

Rb1-inducible coiled-coil 1 (Rb1cc1) is a DNA-binding protein and is abundantly expressed in human musculoskeletal cells.[15] Rb1cc1 expressed at high levels is associated with the maturation of human embryonic musculoskeletal cells, thus, Rb1cc1 is prerequisite for myogenic differentiation.[16]

EIF3J:

EIF3J encodes for eukaryotic translation initiation factor 3 subunit J, which is a core subunit of the eukaryotic initiation factor 3 complex participating in the initiation of translation by aiding in the recruitment of protein and mRNA components to the 40S ribosome. (RefSeq, Sep 2013) It is highly expressed in skeletal muscle[17] but its potential impact on muscle function is not clear.

Supplementary Note

Sensitivity analysis of PTV-burden associations with hand grip strength

We had conducted several sensitivity analyses to assess the potential impacts of additional disease outcomes and skeletal muscle mass on our findings for hand grip strength. First, we had further excluded any participants with Osteoarthritis including Rhizarthrosis (self-reported or ICD-10: M15-M19; N = 34,882), Rheumatoid arthritis (self-reported or ICD-10: M05-M06; N = 3,960), Osteoporosis (self-reported or ICD-10: M80-M81; N = 5,723), Dupuytren's contracture (self-reported or ICD-10: M720; N = 1,284). Disease diagnosis was defined using both in-patient Hospital Episode Statistics (UKB fieldfields 41270 and 41280) and self-reported data at baseline (UKB fieldfields 20002 and 20008). The results were shown in Supplementary Data 17 and consistent findings were observed compared with our main analysis. Furthermore, we also excluded any cancer patients (N = 28,243) in addition to the aforementioned diseases and observed similar findings, ruling out the potential impact of cancer on our top findings for muscle strength (Supplementary Data 18). Another sensitivity analysis was conducted to assess the impact of further adjustment of skeletal muscle mass on our findings for hand grip strength. After including whole-body lean mass (UKB field 23101) as an additional covariate, we observed consistent findings with our main analysis, indicating that the hand grip strength genes we found were not driven by change in skeletal muscle mass.

Skeletal muscle isoform-specific analysis of genes with significant PTV-burden associations with hand grip strength

In our main analysis, all PTV and missense variant impact were predicted on the canonical transcript. To verify the robustness of such approach for our top findings when the primary transcript expressed in skeletal muscle is different than the canonical transcript, we referenced isoform expression data in the skeletal muscle tissue from the GTEx v8 and found three genes (*TTN*, *OBSCN* and *RB1CC1*) that showed either a non-canonical isoform as the primary skeletal muscle isoform (*TTN* and *OBSCN*) or relatively high skeletal muscle abundance of a non-canonical isoform (*RB1CC1*). We re-mapped PTVs to these isoforms and tested associations with hand grip strength (Supplementary Data 9) and found consistent results for *TTN* and *RB1CC1*. Regarding *OBSCN*, we identified a reduction in significance level ($p = 6 \times 10^{-4}$) but consistent directionality in one of the two skeletal muscle isoforms tested (ENST00000636476) and non-significant results ($p = 0.11$) for the other isoform (ENST00000474237). However, the latter isoform is coded by a much shorter transcript with the majority of PTVs mapped as unaffected.

Supplementary Figure 1. Phenome-wide association of *TTN* PTV-burden in the UK Biobank. 3,654 binary and 238 quantitative phenotypes were tested, red dashed line indicates the Bonferroni-corrected significance threshold. Top nine associations were annotated. Source data are provided in Supplemental Data 8.

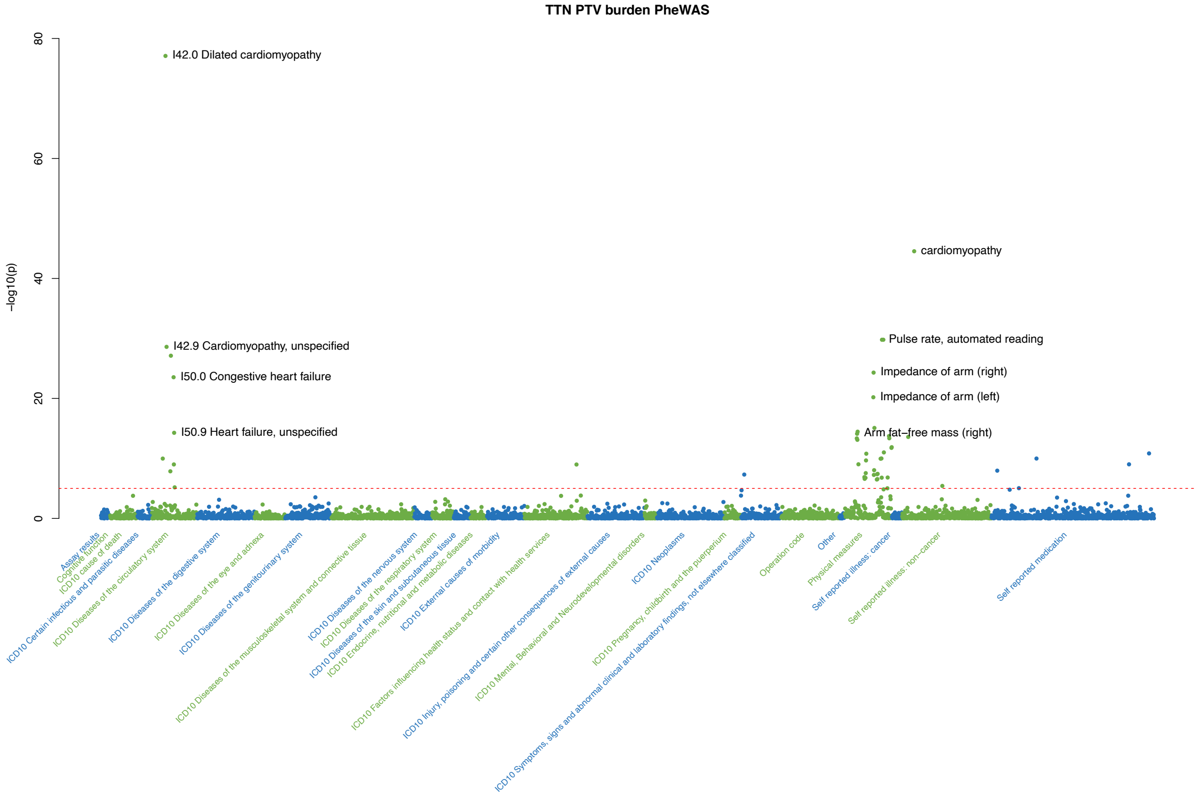

Supplementary Figure 2. Single PTV association of *OBSCN* with HGS in the UK Biobank. Effect sizes of each PTV in *OBSCN* were plotted against its genomic position, with exon numbers and protein domains demonstrated at the bottom. ClinVar pathogenic/likely pathogenic PTVs were colored as red diamond. Pathogenic LoF variants recently reported for severe recurrent rhabdomyolysis[4] were highlighted in dark red. Source data are provided in the Source Data file.

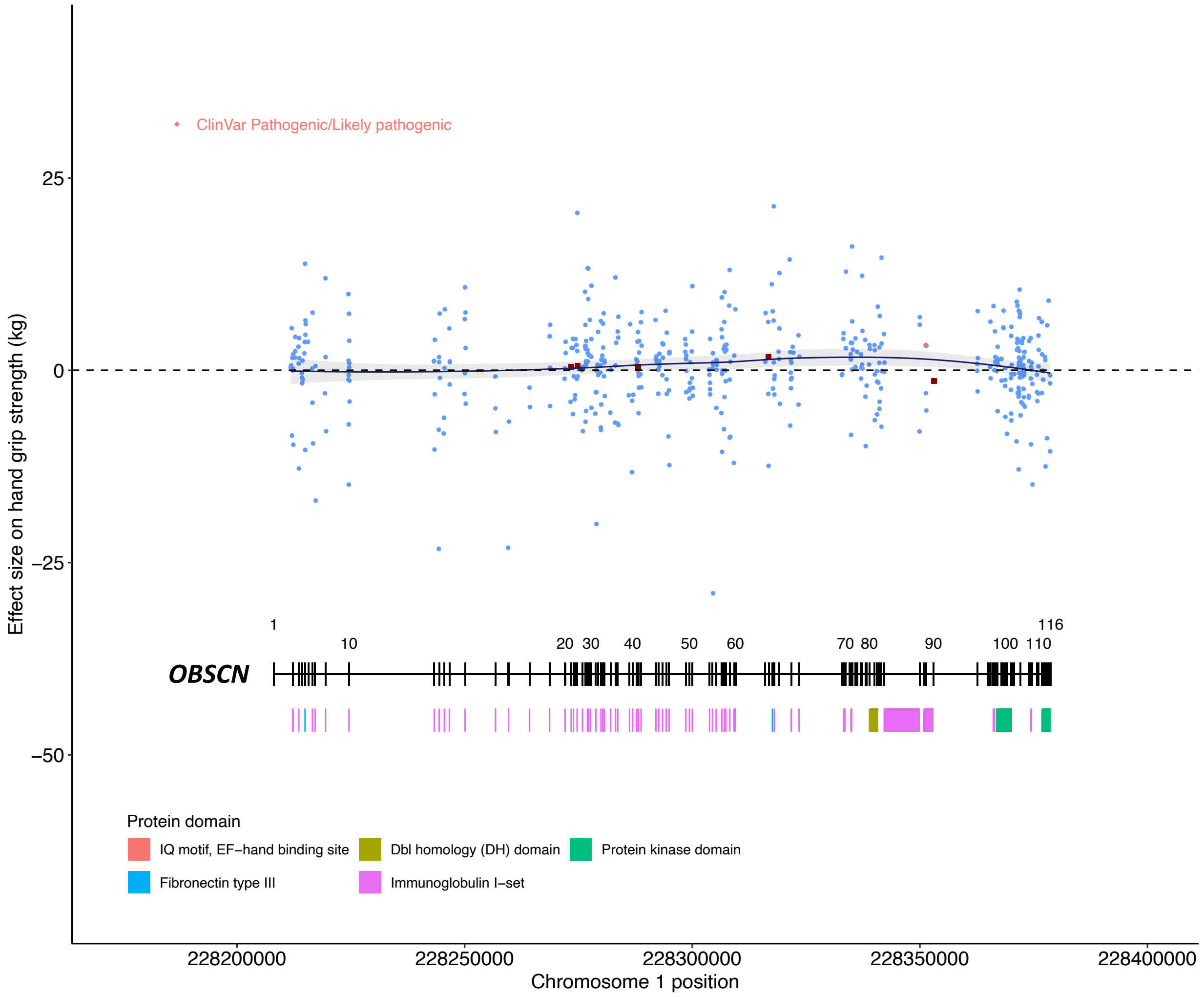

Supplementary Figure 3. Forelimb grip strength (FGS, unnormalized) of heterozygous (HET) and homozygous (HOM) Kdm5b mutant mice vs. wild type (WT) in N=55 biologically independent animals. FGS was corrected for cohort effects. Z-scores calculated from residuals were compared across different genotypes. P-values were derived from two-sided t-tests. *P=0.002. Source data are provided in the Source Data file. center line: median; box limits: upper and lower quartiles; whiskers: minimum or lower quartile minus 1.5 times IQR and maximum or upper quartile plus 1.5 times IQR.

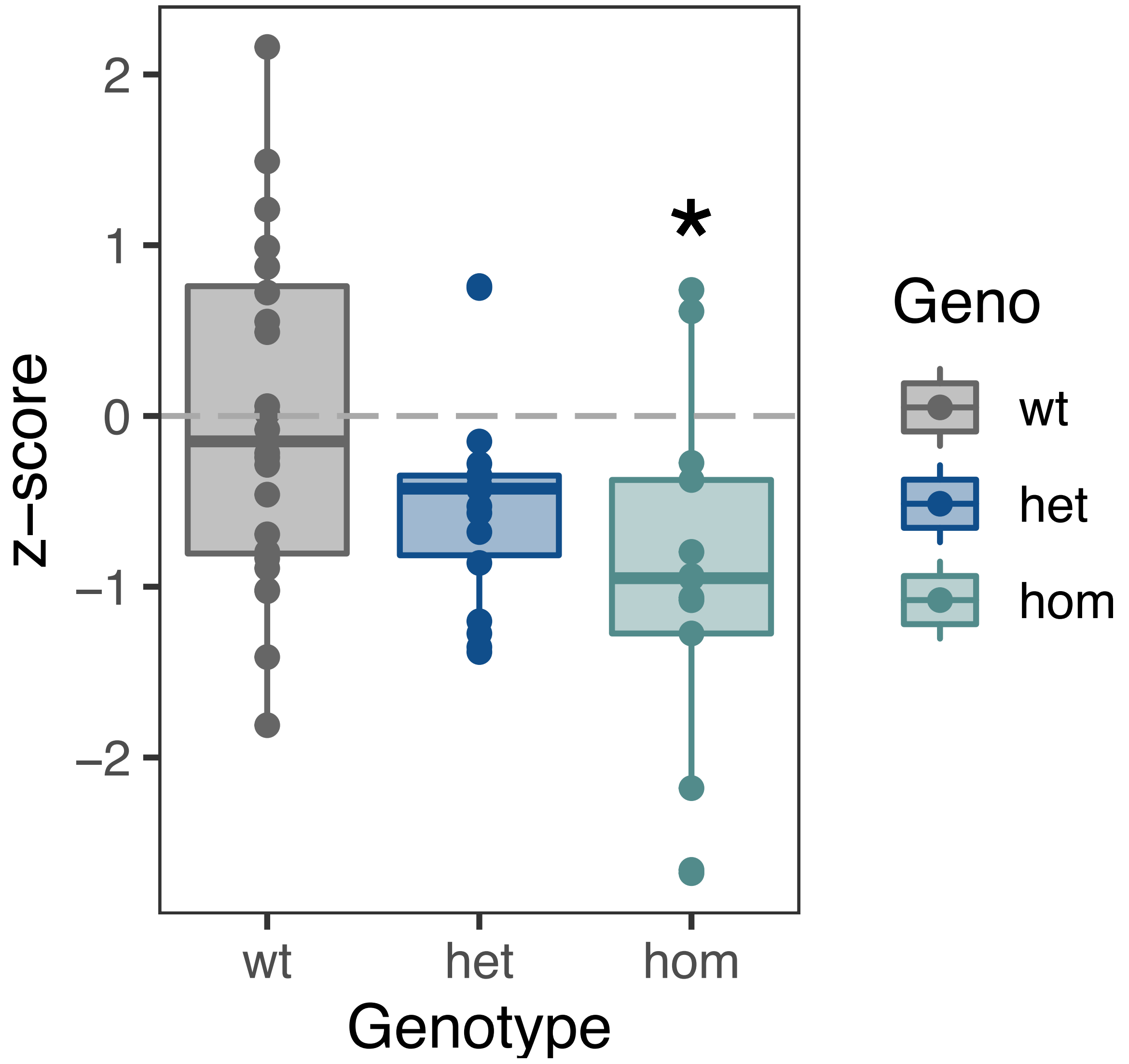

Supplementary Figure 4. Correlation of hand grip strength with standing height in humans and forelimb grip strength with femur length in mice. Simple linear regression line was plotted. Pearson's correlation coefficient and p-value were shown. P-values were derived from two-sided t-tests. Source Data are provided in the Source Data file.

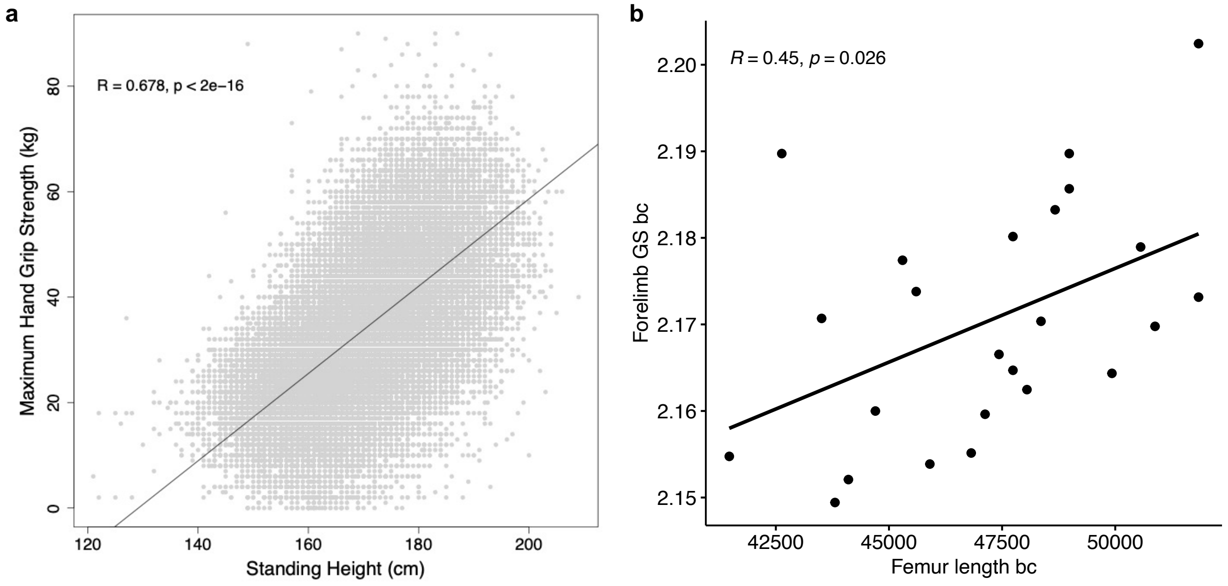

Supplementary Figure 5. Tissue-expressed gene-set PTV-burden associations with hand grip strength. Gene sets were constructed based on 10,992 genes that showed elevated expression in at least 1 of 36 different tissues from Human Protein Atlas. Number of genes, effect size of PTV-burden on hand grip strength and false discovery rate (FDR) were shown for each tissue-expressed gene set. Source data are provided in the Source Data file.

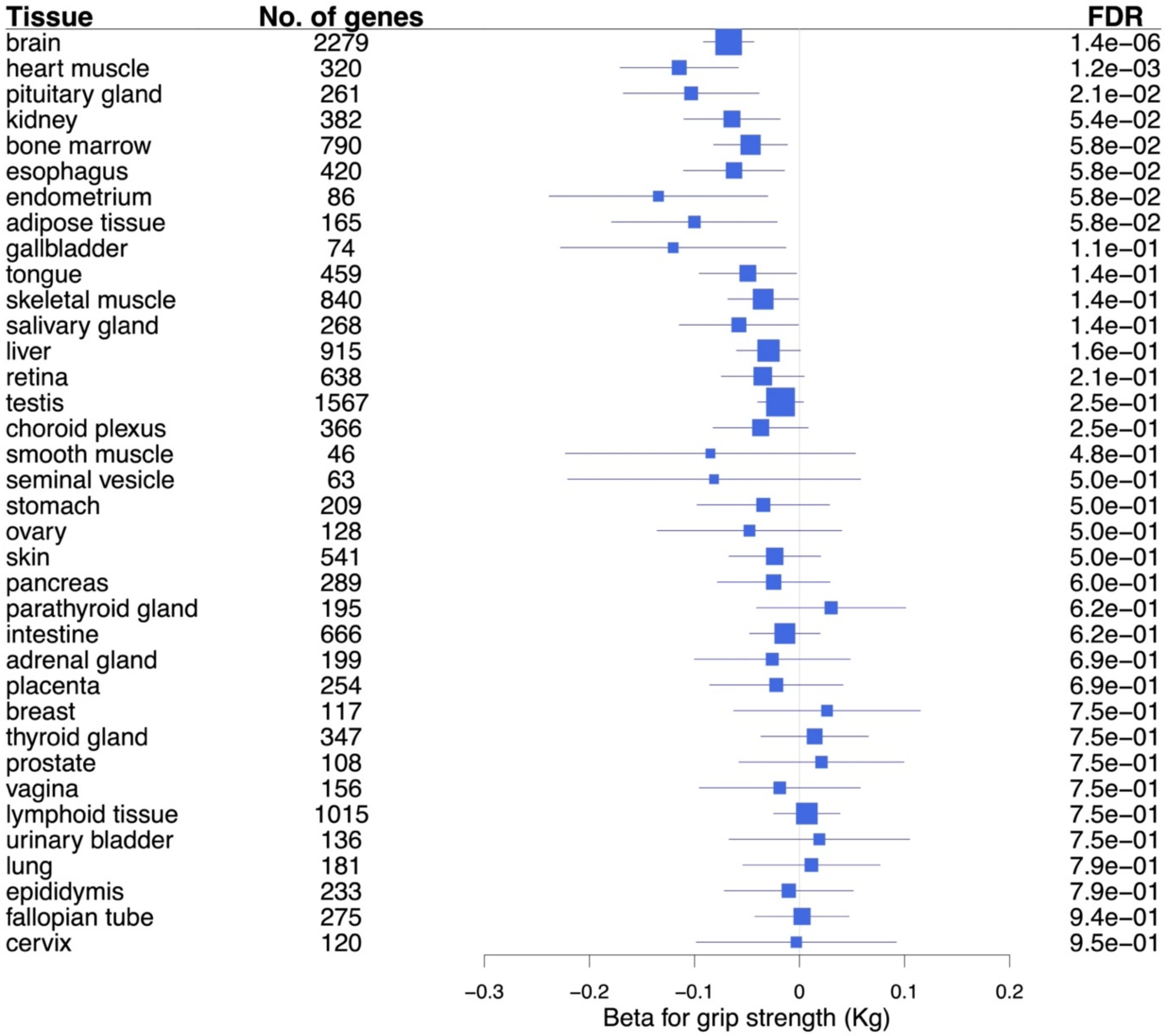

Supplementary Figure 6. Polygenic risk score (PRS) effects on hand grip strength (HGS) stratified for PTV carrier status of Mendelian neuromuscular diseases (NMDs). Mean residualized HGS was plotted against percentiles of HGS-PRS for carriers of PTVs in autosomal dominant NMD genes, carriers of PTVs in autosomal recessive NMD genes, and non-carriers. Linear regression line was derived for each group and plotted in corresponding colors. Shaded area in gray indicates 95% confidence interval. Source data are provided in the Source Data file.

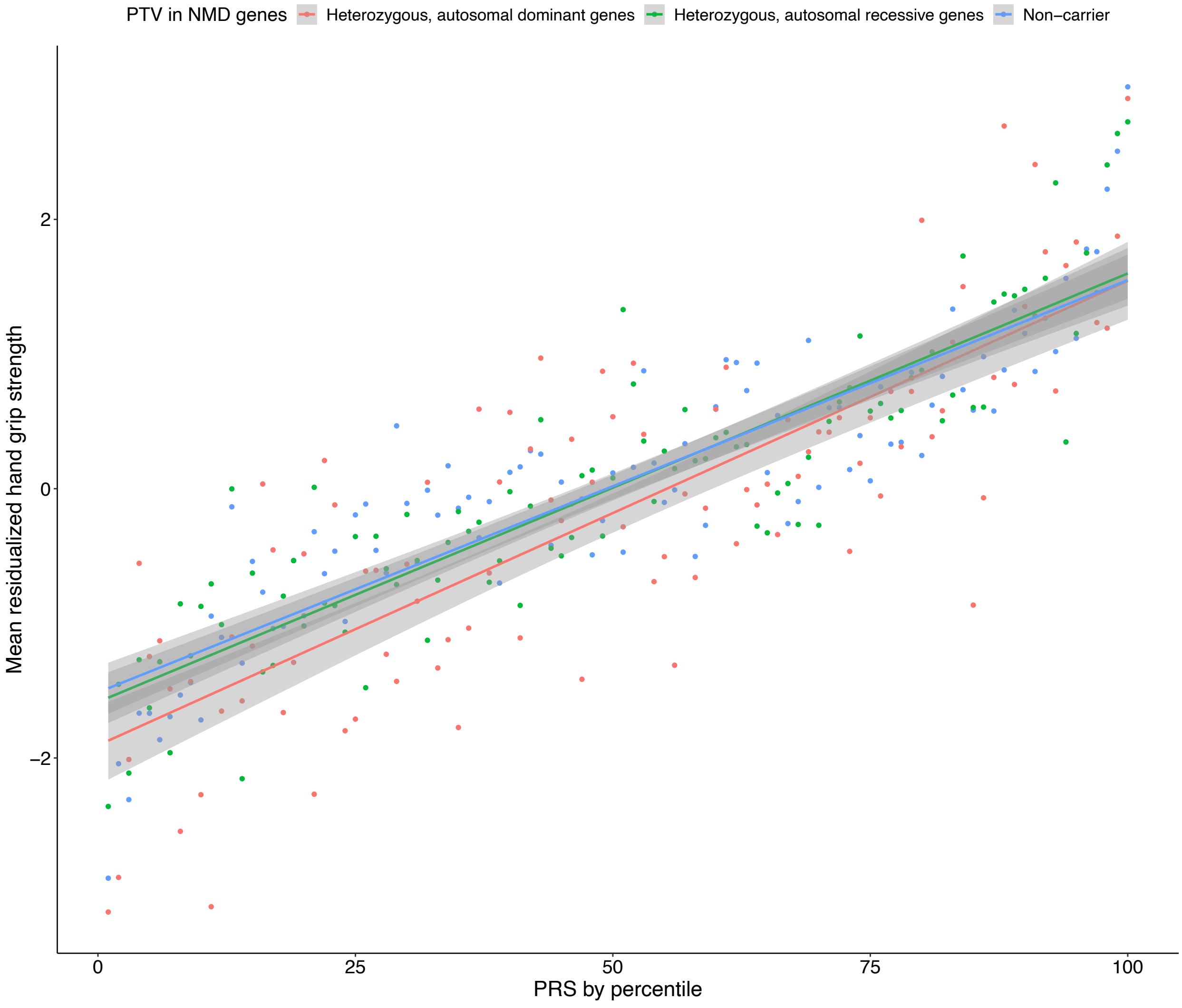

Supplementary References

1 Benian, G. M. & Mayans, O. Titin and obscurin: giants holding hands and discovery of a new Ig domain subset. *J Mol Biol* **427**, 707-714, doi:10.1016/j.jmb.2014.12.017 (2015).

2 Randazzo, D., Pierantozzi, E., Rossi, D. & Sorrentino, V. The potential of obscurin as a therapeutic target in muscle disorders. *Expert Opin Ther Targets* **21**, 897-910, doi:10.1080/14728222.2017.1361931 (2017).

3 Wu, G. *et al.* Truncating Variants in OBSCN Gene Associated With Disease-Onset and Outcomes of Hypertrophic Cardiomyopathy. *Circ Genom Precis Med* **14**, e003401, doi:10.1161/CIRCGEN.121.003401 (2021).

4 Cabrera-Serrano, M. *et al.* Bi-allelic loss-of-function OBSCN variants predispose individuals to severe recurrent rhabdomyolysis. *Brain*, doi:10.1093/brain/awab484 (2021).

5 Blair, L. P., Cao, J., Zou, M. R., Sayegh, J. & Yan, Q. Epigenetic Regulation by Lysine Demethylase 5 (KDM5) Enzymes in Cancer. *Cancers (Basel)* **3**, 1383-1404, doi:10.3390/cancers3011383 (2011).

6 Huang, P. H. *et al.* Histone deacetylase inhibitors stimulate histone H3 lysine 4 methylation in part via transcriptional repression of histone H3 lysine 4 demethylases. *Mol Pharmacol* **79**, 197-206, doi:10.1124/mol.110.067702 (2011).

7 Tian, H. *et al.* Role of Histone Deacetylases in Skeletal Muscle Physiology and Systemic Energy Homeostasis: Implications for Metabolic Diseases and Therapy. *Front Physiol* **11**, 949, doi:10.3389/fphys.2020.00949 (2020).

8 Backe, M. B. *et al.* The Lysine Demethylase KDM5B Regulates Islet Function and Glucose Homeostasis. *J Diabetes Res* **2019**, 5451038, doi:10.1155/2019/5451038 (2019).

9 Wang, Q. *et al.* Rare variant contribution to human disease in 281,104 UK Biobank exomes. *Nature* **597**, 527-532, doi:10.1038/s41586-021-03855-y (2021).

10 Faundes, V. *et al.* Histone Lysine Methylases and Demethylases in the Landscape of Human Developmental Disorders. *Am J Hum Genet* **102**, 175-187, doi:10.1016/j.ajhg.2017.11.013 (2018).

11 Firth, J. *et al.* Grip Strength Is Associated With Cognitive Performance in Schizophrenia and the General Population: A UK Biobank Study of 476559 Participants. *Schizophr Bull* **44**, 728-736, doi:10.1093/schbul/sby034 (2018).

12 Zhou, T. *et al.* Modulation of IGF1R Signaling Pathway by GIGYF1 in High Glucose-Induced SHSY-5Y Cells. *DNA Cell Biol* **37**, 1044-1054, doi:10.1089/dna.2018.4336 (2018).

13 Morissette, M. R., Cook, S. A., Buranasombati, C., Rosenberg, M. A. & Rosenzweig, A. Myostatin inhibits IGF-I-induced myotube hypertrophy through Akt. *Am J Physiol Cell Physiol* **297**, C1124-1132, doi:10.1152/ajpcell.00043.2009 (2009).

14 Deaton, A. M. *et al.* Gene-level analysis of rare variants in 379,066 whole exome sequences identifies an association of GIGYF1 loss of function with type 2 diabetes. *Sci Rep* **11**, 21565, doi:10.1038/s41598-021-99091-5 (2021).

15 Chano, T. *et al.* Identification of RB1CC1, a novel human gene that can induce RB1 in various human cells. *Oncogene* **21**, 1295-1298, doi:10.1038/sj.onc.1205178 (2002).

16 Watanabe, R. *et al.* Rb1cc1 is critical for myoblast differentiation through Rb1 regulation. *Virchows Arch* **447**, 643-648, doi:10.1007/s00428-004-1183-1 (2005).

17 Uhlén, M. *et al.* Proteomics. Tissue-based map of the human proteome. *Science* **347**, 1260419, doi:10.1126/science.1260419 (2015).
