# Supplementary material for: Rare genetic variants impact muscle strength
Source: Nat Commun. 2023 Jun 10;14:3449. doi: 10.1038/s41467-023-39247-1 (PMC10257725; doi:10.1038/s41467-023-39247-1)
Supplement: Supplementary file 2 — Description of Additional Supplementary Files [file 41467_2023_39247_MOESM2_ESM.pdf]

Description of Additional Supplementary Files

File Name: Supplementary Data 1
Description: PTV and missense-burden associations with hand grip strength aggregating LoF intolerant genes, LoF tolerant genes, and all genes. Rare coding variants were annotated by functional consequences into protein-truncating, missense and synonymous (negative control), then aggregated over protein coding genes by pLI (v2.1.1), into LoF intolerant (pLI ≥ 0.9) set, LoF tolerant (pLI < 0.9) set and all gene set to construct global rare coding variant burden. Rare variant burden associations with hand grip strength were tested using linear regression models. P-values were derived from two-sided t-tests of linear regression coefficients. A Bonferroni correction was used to adjust for multiple testing. Significant associations were highlighted in red. Beta: beta coefficient for hand grip strength; SE: standard error for beta; P: p-value for beta; Bonf.P: Bonferroni-adjusted p-values.

File Name: Supplementary Data 2
Description: Gene-level PTV-burden associations with hand grip strength in the UK Biobank (filtered on MAF<0.001 and number of PTV carriers >= 10). Gene-level protein-truncating variant burden was constructed by aggregating all PTVs with MAF<0.001 for each protein-coding gene. Genes with >=10 PTV carriers were included in the hand grip strength association analysis, conducted using linear regression models with P-values derived from two-sided t-tests of regression coefficients. A Bonferroni correction was used to adjust for multiple testing. A sex-stratified analysis was also conducted in males and females separately. GENE: gene symbol; CHR: chromosome; BP: base-pair location; pLI: pLI score of gene; N: total sample size; beta: beta coefficient for hand grip strength; se: standard error for beta; P: p-value for beta; N_carrier: number of PTV carriers; Bonf: Bonferroni-adjusted p-values; FDR: false discovery rate; N.male: sample size for male-only analysis; beta.male: beta coefficient for hand grip strength in male-only analysis; se.male: standard error for beta in male-only analysis; P.male: p-value for beta in male-only analysis; N_carrier.male: number of PTV carriers in male-only analysis; N.female: sample size for female-only analysis; beta.female: beta coefficient for hand grip strength in female-only analysis; se.female: standard error for beta in female-only analysis; P.female: p-value for beta in female-only analysis; N_carrier.female: number of PTV carriers in female-only analysis.

File Name: Supplementary Data 3
Description: Gene-level missense-burden (CADD > 30) associations with hand grip strength in the UK Biobank (filtered on MAF<0.001 and number of PTV carriers >= 10). Gene-level missense variant burden was constructed by aggregating all missense variants with MAF<0.001 and CADD>30 for each protein-coding gene. Genes with >=10 missense variant carriers were included in the hand grip strength association analysis, conducted using linear regression models with P-values derived from two-sided t-tests of regression coefficients. A Bonferroni correction was used to adjust for multiple testing. GENE: gene symbol; N: total sample size; N_carrier: number of missense variant carriers; beta: beta coefficient for hand grip strength; se: standard error for beta; P: p-value for beta; Bonf: Bonferroni-adjusted p-values; FDR: false discovery rate.

File Name: Supplementary Data 4
Description: Gene-level missense-burden (CADD: 20-30) associations with hand grip strength in the UK Biobank (filtered on MAF<0.001 and number of PTV carriers >= 10). Gene-level missense variant burden was constructed by aggregating all missense variants with MAF<0.001 and CADD between 20 to 30 for each protein-coding gene. Genes with >=10 missense variant carriers were included in the hand grip strength association analysis, conducted using linear regression models with P-values derived from two-sided t-tests of regression coefficients. A Bonferroni correction was used to adjust for multiple testing. GENE: gene symbol; N: total sample size; N_carrier: number of missense variant carriers; beta: beta coefficient for hand grip strength; se: standard error for beta; P: p-value for beta; Bonf: Bonferroni-adjusted p-values; FDR: false discovery rate.

File Name: Supplementary Data 5
Description: Gene-level PTV-burden associations with hand grip strength in the UK Biobank for chromosome X (filtered on MAF<0.001 and number of PTV carriers >= 10). Gene-level protein-truncating variant burden was constructed by aggregating all PTVs with MAF<0.001 for each protein-coding gene on the X chromosome. Genes with >=10 PTV carriers were included in the hand grip strength association analysis, conducted using linear regression models with P-values derived from two-sided t-tests of regression coefficients. A Bonferroni correction was used to adjust for multiple testing. A sex-stratified analysis was also conducted in males and females separately. GENE: gene symbol; N: total sample size; beta: beta coefficient for hand grip strength; se: standard error for beta; P: p-value for beta; N_carrier: number of PTV carriers; N.male: sample size for male-only analysis; beta.male: beta coefficient for hand grip strength in male-only analysis; se.male: standard error for beta in male-only analysis; P.male: p-value for beta in male-only analysis; N_carrier.male: number of PTV carriers in male-only analysis; N.female: sample size for female-only analysis; beta.female: beta coefficient for hand grip strength in female-only analysis; se.female: standard error for beta in female-only analysis; P.female: p-value for beta in female-only analysis; N_carrier.female: number of PTV carriers in female-only analysis.

File Name: Supplementary Data 6
Description: Gene-level missense-burden (CADD > 30) associations with hand grip strength in the UK Biobank for chromosome X (filtered on MAF<0.001 and number of PTV carriers >= 10). Gene-level missense variant burden was constructed by aggregating all missense variants with MAF<0.001 and CADD>30 for each protein-coding gene on the X chromosome. Genes with >=10 missense variant carriers were included in the hand grip strength association analysis, conducted using linear regression models with P-values derived from two-sided t-tests of regression coefficients. A Bonferroni correction was used to adjust for multiple testing. GENE: gene symbol; N: total sample size; beta: beta coefficient for hand grip strength; se: standard error for beta; P: p-value for beta; N_carrier: number of missense variant carriers.

File Name: Supplementary Data 7
Description: Gene-level missense-burden (CADD 20-30) associations with hand grip strength in the UK Biobank for chromosome X (filtered on MAF<0.001 and number of PTV carriers >= 10). Gene-level missense variant burden was constructed by aggregating all missense variants with MAF<0.001 and CADD between 20 to 30 for each protein-coding gene on the X chromosome.

Genes with >=10 missense variant carriers were included in the hand grip strength association analysis, conducted using linear regression models with P-values derived from two-sided t-tests of regression coefficients. A Bonferroni correction was used to adjust for multiple testing. GENE: gene symbol; N: total sample size; beta: beta coefficient for hand grip strength; se: standard error for beta; P: p-value for beta; N_carrier: number of missense variant carriers.

File Name: Supplementary Data 8
Description: TTN PTV-burden phenome-wide association analysis results in the UK Biobank. Associations with PTV-burden of TTN were tested for 3,654 binary and 238 quantitative phenotypes. For binary phenotype, a Firth logistic regression was run for those with significant associations (P<0.01) identified using regular logistic regressions. Linear regression models were used for quantitative phenotypes. Wald-tests were used to derive p-values for logistic regression coefficients as two-sided t-tests for linear regression coefficients. A Bonferroni correction was used to adjust for multiple testing. Phenotype: phenotype tested; Category: phenotype category; Gene: gene symbol; Beta: beta coefficient for phenotype; P: p-value for beta; Model: model used for phenotype.

File Name: Supplementary Data 9
Description: PTV-burden associations of significant genes with hand grip strength remapped to the primary transcripts (if different than canonical) in skeletal muscle. Of the six genes (*KDM5B*, *OBSCN*, *GIGYF1*, *TTN*, *RB1CC1* and *EIF3J*) with significant PTV-burden associations with hand grip strength for their canonical transcript, three (*OBSCN*, *TTN*, *RB1CC1*) were found to have a non-canonical isoform as the primary skeletal muscle isoform (*TTN* and *OBSCN*) or relatively high skeletal muscle abundance of a non-canonical isoform (*RB1CC1*) based on isoform expression data in the skeletal muscle tissue from the GTEx v8. PTv-burden associations of these non-canonical isoforms were tested for hand grip strength using linear regression models with P-values derived from two-sided t-tests of regression coefficients. Results were shown for both canonical and non-canonical isoforms tested. Gene: gene symbol; Ensembl ID (GTEx TPM): Ensembl ID for transcript and expression level in GTEx skeletal muscle tissue by transcript per million; N: total sample size; beta: beta coefficient for hand grip strength; se: standard error for beta; P: p-value for beta; N_carrier: number of PTV carriers.

File Name: Supplementary Data 10
Description: Associations of single PTV in TTN with hand grip strength in the UK Biobank. Single PTV-associations of TTN with hand grip strength were tested using linear regression models with P-values derived from two-sided t-tests of regression coefficients. Variant_hg38: variant ID in the format of chromosome: base pair location (genome build hg38): reference allele: alternative allele; chr_hg19: chromosome; pos_hg19: base pair location (genome build hg19); gene: gene symbol; type: VEP annotated variant consequence; amino acid: VEP annotated amino acid change due to variant; N: total sample size; beta: beta coefficient for hand grip strength; se: standard error for beta; P: p-value for beta; N_carrier: number of PTV carriers; ClinVar P/LP: variant included as pathogenic/likely pathogenic by ClinVar; CLNDN: clinical phenotype associated with variant in ClinVar.

File Name: Supplementary Data 11
Description: Common (MAF>0.01) variant associations with hand grip strength reported in GWAS Catalog. A total of 269 associations reported for "grip strength measurement" in the GWAS Catalog (https://www.ebi.ac.uk/gwas/efotraits/EFO_0006941) were extracted and filtered to 236 associations with minor allele frequency > 0.01. 1,787 protein-coding genes within a +/- 500kb window of these reported lead variants were annotated for these common variant loci. Variant: lead common variant; Risk allele: effect allele; P-value: GWAS Catalog p-value; RAF: effect allele frequency; Beta: beta coefficient; CI: 95% confidence interval; Reported trait: trait reported in GWAS Catalog; Study accession: study ID in GWAS Catalog; Location (hg38): chromosomal location; Protein-coding genes (+/-500kb): protein-coding genes annotated for lead variant.

File Name: Supplementary Data 12
Description: PTV-burden associations with hand grip strength for genes within 500kb of common (MAF>0.01) variants associated with hand grip strength reported in GWAS Catalog. For genes within +/- 500kb of common (MAF>0.01) variant associations with hand grip strength reported in GWAS Catalog, PTV-burden associations with hand grip strength were tested using linear regression models with p-values derived by two-sided t-tests of regression coefficients. GENE: gene symbol; CHR: chromosome; BP: base-pair location; N: total sample size; beta: beta coefficient for hand grip strength; se: standard error for beta; P: p-value for beta; N_carrier: number of PTV carriers; Bonf: Bonferroni-adjusted p-values; FDR: false discovery rate.

File Name: Supplementary Data 13
Description: Pathway-based PTV-burden associations with hand grip strength. Pathway-based gene-set PTV-burden was assessed in a self-contained manner by summing the rare alleles of PTVs of all genes in each pathway gene-set, association testing was then carried out using linear regression models. P-values were derived by two-sided t-tests of regression coefficients. A false discovery rate < 0.05 was used to declare statistical significance. Pathway: pathway gene-set tested; N: total sample size; beta: beta coefficient for hand grip strength; se: standard error for beta; P: p-value for beta; source: source of pathway gene-set; FDR: false discovery rate.

File Name: Supplementary Data 14
Description: Gene-level PTV-burden associations with hand grip strength in the UK Biobank adjusted for education or reaction time (filtered on MAF<0.001 and number of PTV carriers >= 10). Sensitivity analyses of gene-level PTV-burden associations with hand grip strength were conducted by further adjusting for years of schooling or reaction time in the UK Biobank. Gene-level PTV-burden was constructed by aggregating all PTVs with MAF<0.001 for each protein-coding gene. Genes with >=10 PTV carriers were included in the hand grip strength association analysis, conducted using linear regression models with P-values derived from two-sided t-tests of regression coefficients. GENE: gene symbol; CHR: chromosome; BP: base-pair location; N: total sample size; beta: beta coefficient for hand grip strength; se: standard error for beta; P: p-value for beta; N_carrier: number of PTV carriers; Bonf: Bonferroni-adjusted p-values; FDR: false discovery rate; N.edu_adj: total sample size for education-adjusted analysis; beta.edu_adj: beta coefficient for hand grip strength in education-adjusted analysis; se.edu_adj: standard error for beta in education-adjusted analysis; P.edu_adj: p-value for beta in education-adjusted analysis; N. rt_adj: total sample size for reaction time-adjusted analysis; beta. rt_adj: beta

coefficient for hand grip strength in reaction time-adjusted analysis; se. rt_adj: standard error for beta in reaction time-adjusted analysis; P. rt_adj: p-value for beta in reaction time-adjusted analysis.

File Name: Supplementary Data 15
Description: 199 Mendelian neuromuscular disease genes included in the Invitae Comprehensive NMD panel. PTV carriers for a list of 199 Mendelian neuromuscular disease genes referenced from the Invitae Comprehensive NMD panel were identified in the UK Biobank. Gene: gene symbol; ENSG: Ensembl gene ID; MIM_gene_number: gene ID from Online Mendelian Inheritance in Man (https://www.omim.org/); Inheritance: inheritance model from Online Mendelian Inheritance in Man database.

File Name: Supplementary Data 16
Description: Interaction analysis of PRS for hand grip strength with rare PTV (MAF<0.001) for Mendelian neuromuscular diseases. PRS was constructed for 38,118 carriers of PTVs in 199 genes associated with Mendelian neuromuscular diseases (Supplementary Data 15) as well as 38,118 randomly selected non-carriers. Interaction between PRS and PTV-burden for Mendelian neuromuscular disease genes were assessed using a linear regression model with p-values derived from two-sided t-tests of regression coefficients. Regression models for an additive effect as well as the interaction test were shown. Beta coefficients (Estimate), standard errors (SE), and p-values (P) for PRS, PTV-burden of autosomal dominant genes (PTV_AD), PTV-burden of autosomal recessive genes (PTV_AR) were shown for the additive effect model. Beta coefficients (Estimate), standard errors (SE), and p-values (P) additionally for the interaction between PRS and PTV_AD (PRS * PTV_AD) as well as the interaction between PRS and PTV_AR (PRS * PTV_AR) were shown for the interaction model.

File Name: Supplementary Data 17
Description: Gene-level PTV-burden associations with hand grip strength in the UK Biobank (filtered on MAF<0.001 and number of PTV carriers >= 10,
sensitivity analysis that further excluded participants with diagnosis of Osteoarthritis, Rheumatoid arthritis, Rhizarthrosis, Osteoporosis, Dupuytren's contracture). A sensitivity analysis for gene-level PTV-burden associations with hand grip strength was conducted by further excluding any participants with Osteoarthritis including Rhizarthrosis (self-reported or ICD-10: M15-M19; N = 34,882), Rheumatoid arthritis (self-reported or ICD-10: M05-M06; N = 3,960), Osteoporosis (self-reported or ICD-10: M80-M81; N = 5,723), Dupuytren's contracture (self-reported or ICD-10: M720; N = 1,284). Association analysis was conducted using linear regression models with p-values derived from two-sided t-tests of regression coefficients. GENE: gene symbol; N: total sample size; beta: beta coefficient for hand grip strength; se: standard error for beta; P: p-value for beta; N_carrier: number of PTV carriers.

File Name: Supplementary Data 18
Description: Gene-level PTV-burden associations with hand grip strength in the UK Biobank (filtered on MAF<0.001 and number of PTV carriers >= 10,
sensitivity analysis that further excluded participants with diagnosis of Osteoarthritis, Rheumatoid arthritis, Rhizarthrosis, Osteoporosis, Dupuytren's contracture, and cancer). A sensitivity analysis for gene-level PTV-burden associations with hand grip strength was

conducted by further excluding any participants with Osteoarthritis including Rhizarthrosis (self-reported or ICD-10: M15-M19; N = 34,882), Rheumatoid arthritis (self-reported or ICD-10: M05-M06; N = 3,960), Osteoporosis (self-reported or ICD-10: M80-M81; N = 5,723), Dupuytren's contracture (self-reported or ICD-10: M720; N = 1,284) and cancer (N = 28,243). Association analysis was conducted using linear regression models with p-values derived from two-sided t-tests of regression coefficients. GENE: gene symbol; N: total sample size; beta: beta coefficient for hand grip strength; se: standard error for beta; P: p-value for beta; N_carrier: number of PTV carriers.

File Name: Supplementary Data 19
Description: Gene-level PTV-burden associations with hand grip strength in the UK Biobank (filtered on MAF<0.001 and number of PTV carriers >= 10, sensitivity analysis that further adjusted for whole body lean mass). A sensitivity analysis for gene-level PTV-burden associations with hand grip strength was conducted by further adjusting for whole body lean mass. Association analysis was conducted using linear regression models with p-values derived from two-sided t-tests of regression coefficients. GENE: gene symbol; N: total sample size; beta: beta coefficient for hand grip strength; se: standard error for beta; P: p-value for beta; N_carrier: number of PTV carriers.
