# Supplementary material for: Rare genetic variants impact muscle strength
Source: Nat Commun. 2023 Jun 10;14:3449. doi: 10.1038/s41467-023-39247-1 (PMC10257725; doi:10.1038/s41467-023-39247-1)
Supplement: Supplementary file 4 — Reporting Summary [file 41467_2023_39247_MOESM4_ESM.pdf]

nature portfolio

Corresponding author(s): Yunfeng Huang, Heiko Runz

Last updated by author(s): Apr 3, 2023

Reporting Summary

Nature Portfolio wishes to improve the reproducibility of the work that we publish. This form provides structure for consistency and transparency in reporting. For further information on Nature Portfolio policies, see our Editorial Policies and the Editorial Policy Checklist.

Statistics

For all statistical analyses, confirm that the following items are present in the figure legend, table legend, main text, or Methods section.

n/a	Confirmed	
☐	☒	The exact sample size (*n*) for each experimental group/condition, given as a discrete number and unit of measurement
☐	☒	A statement on whether measurements were taken from distinct samples or whether the same sample was measured repeatedly
☐	☒	The statistical test(s) used AND whether they are one- or two-sided<br>*Only common tests should be described solely by name; describe more complex techniques in the Methods section.*
☐	☒	A description of all covariates tested
☐	☒	A description of any assumptions or corrections, such as tests of normality and adjustment for multiple comparisons
☐	☒	A full description of the statistical parameters including central tendency (e.g. means) or other basic estimates (e.g. regression coefficient) AND variation (e.g. standard deviation) or associated estimates of uncertainty (e.g. confidence intervals)
☐	☒	For null hypothesis testing, the test statistic (e.g. *F*, *t*, *r*) with confidence intervals, effect sizes, degrees of freedom and *P* value noted<br>*Give P values as exact values whenever suitable.*
☒	☐	For Bayesian analysis, information on the choice of priors and Markov chain Monte Carlo settings
☒	☐	For hierarchical and complex designs, identification of the appropriate level for tests and full reporting of outcomes
☐	☒	Estimates of effect sizes (e.g. Cohen's *d*, Pearson's *r*), indicating how they were calculated

Our web collection on statistics for biologists contains articles on many of the points above.

Software and code

Policy information about availability of computer code

Data collection	No software was used.
Data analysis	Variant annotation was done using Variant Effect Predictor (VEP) v96; GWAS was run using regenie (v2.0.1); MAGMA (v1.6) from FUMA (v1.3.6); susieR (v0.11.7); Polygenic risk score was constructed using PRS-CS (v1.0.0); MASS (v7.3-57); All other statistical analyses were done using R (3.6.1) Computational codes used for analyses in the present study are available under the following link: https://github.com/yunfenghuang618/HGS_exome_paper

For manuscripts utilizing custom algorithms or software that are central to the research but not yet described in published literature, software must be made available to editors and reviewers. We strongly encourage code deposition in a community repository (e.g. GitHub). See the Nature Portfolio guidelines for submitting code & software for further information.

Data

Policy information about availability of data

All manuscripts must include a data availability statement. This statement should provide the following information, where applicable:

- Accession codes, unique identifiers, or web links for publicly available datasets
- A description of any restrictions on data availability
- For clinical datasets or third party data, please ensure that the statement adheres to our policy

All phenotypic and genetic data for the UK Biobank are available to researchers under data access request from the UK Biobank. WES data from UK Biobank

participants have been deposited with UK Biobank and are freely available to approved researchers via the UK Biobank Research Analysis Platform (https://www.ukbiobank.ac.uk/enable-your-research/research-analysis-platform). Data used for this study is under application 26041. Summary-level association results produced in the present study are contained in the Supplementary Tables. Source data are provided with this paper. Public datasets used in the present study include: pLoF Metrics (https://storage.googleapis.com/gcp-public-data--gnomad/release/2.1.1/constraint/gnomad.v2.1.1.lof_metrics.by_gene.txt.bgz); Human Protein Atlas (https://www.proteinatlas.org/humanproteome/tissue/tissue+specific); CADD score (https://cadd.gs.washington.edu/download); MSigDB (https://www.gsea-msigdb.org/gsea/msigdb/).

Human research participants

Policy information about studies involving human research participants and Sex and Gender in Research.

Reporting on sex and gender	Sex was imputed using genotype data and adjusted for as a covariate in the analysis. Sex-stratified analysis results were reported when applicable.
Population characteristics	UKB is a UK population study of approximately 500,000 participants aged 40–69 years at recruitment
Recruitment	Assessments were undertaken in 22 centres in Scotland, England and Wales.
Ethics oversight	Analyses in this study were conducted under UK Biobank Approved Project number 26041. Ethic protocols are provided by the UK Biobank Ethics Advisory Committee (https://www.ukbiobank.ac.uk/learn-more-about-uk-biobank/about-us/ethics).

Note that full information on the approval of the study protocol must also be provided in the manuscript.

Field-specific reporting

Please select the one below that is the best fit for your research. If you are not sure, read the appropriate sections before making your selection.

☒ Life sciences ☐ Behavioural & social sciences ☐ Ecological, evolutionary & environmental sciences

For a reference copy of the document with all sections, see nature.com/documents/nr-reporting-summary-flat.pdf

Life sciences study design

All studies must disclose on these points even when the disclosure is negative.

Sample size	Sample size was determined by data availability.
Data exclusions	Out of 409,559 UK Biobank participants of European ancestry (Data-Field 22006), we removed individuals with disease diagnoses that can potentially confound hand grip strength measurements including: COPD (N = 2616), brachial plexus disorders (n=50), or history of injuries in elbow, forearm, wrist and hand (n=7608). Due to the large impact of body size on hand grip strength, we restricted our analysis to samples with non-missing and normal body weight (30 to 200 kg).
Replication	For the kdm5b mutant mice, grip strength experiment was successfully repeated three times for each mouse and the mean value was used. Hand grip strength measurements were taken for both left and right hand per individual in the UK Biobank and the maximum was used for the analysis. No other replication was done in the present study.
Randomization	Randomization is not applicable as this is an observational study.
Blinding	Blinding is not applicable as this is an observational study.

Reporting for specific materials, systems and methods

We require information from authors about some types of materials, experimental systems and methods used in many studies. Here, indicate whether each material, system or method listed is relevant to your study. If you are not sure if a list item applies to your research, read the appropriate section before selecting a response.

Materials & experimental systems

n/a	Involved in the study
☒	☐ Antibodies
☒	☐ Eukaryotic cell lines
☒	☐ Palaeontology and archaeology
☐	☒ Animals and other organisms
☒	☐ Clinical data
☒	☐ Dual use research of concern

Methods

n/a	Involved in the study
☒	☐ ChIP-seq
☒	☐ Flow cytometry
☒	☐ MRI-based neuroimaging

Animals and other research organisms

Policy information about studies involving animals; ARRIVE guidelines recommended for reporting animal research, and Sex and Gender in Research

Laboratory animals	We generated a mouse Kdm5b loss of function allele (MGI:6153378). Breeding of test cohorts was performed on a C57BL/6NJ background. Mice were housed in mixed genotype cages (2-5 mice) with food and water ad-libitum, under controlled temperature and humidity and a 12h light cycle (light on at 7am) at the Research Support Facility of the Wellcome Sanger Institute. At 14-15 weeks of age, 24 wildtype, 18 heterozygous and 13 homozygous Kdm5b mutant male mice were tested.
Wild animals	The study didn't involve wild animals.
Reporting on sex	This information has not been collected.
Field-collected samples	The study didn't involve samples collected from the field.
Ethics oversight	Breeding, housing, and all experimental procedures with mice were approved by the Animal Welfare and Ethical Review Body of the Wellcome Sanger Institute and, conducted under the regulation of UK Home Office license (P6320B89B), and in accordance with institutional guidelines.

Note that full information on the approval of the study protocol must also be provided in the manuscript.
